# Endometritis

**Published:** 1894-07

**Authors:** T. J. Wagley

**Affiliations:** Cleburne, Texas


For Texas Medical Journal.

### ENDOMETRITIS.

BY T. J. WAGLEY, M. D., CLEBURNE, TEXAS.

Read before the Johnson County Medical Society, May 22, 1894.

GENTLEMEN OF THE ASSOCIATION—Having been selected by this Society to read a paper on the subject at the heading of this article, I hope I may be pardoned for including a short notice of areolar hyperplasia, the so-called chronic metritis of some authors. I do this for several reasons: In the first place, the two diseases are very intimately associated, and often co-exist in the same subject; that they are caused chiefly by the same morbid conditions, such as subinvolution, displacements, etc.; are attended by a number of similar symptoms, pain in back and loins, leucorrhœa, menstrual and nervous disorders, etc.; and for the further reason that the treatment of the one necessarily involves that of the other when they happen to occur in the same patient. And I might still add, inasmuch as I am reading to physicians who lay no claims to special knowledge in this branch, the hyperplastic condition of the uterus is sometimes overlooked in this class of cases.

First taking up cervical endometritis, I wish to state the fact that the so-called chronic inflammations, which are not inflammations at all, differ from acute inflammations, inasmuch as the former will often limit their effects to a single membrane, or to a part of an organ, whereas the latter will involve the entire organ.

Thus we have in chronic endocervicitis an affection, not an inflammation, as often described, of the mucous membrane, limited in extent from the os externum to the os internum, perhaps the most frequent of all diseases met with by the gynecologist, due to the frequent exposure of the cervix to irritation during coition, while walking or riding, and as a result of injury during labor.

From the oft-repeated hyperæmic conditions produced by these different irritations, the mucous glands become involved in the morbid action, and secrete a large quantity of viscid mucus, which is found plugging up the os, and is one of the characteristic symptoms of the disease. There are other pathological conditions not necessary to mention, as you have them better described in your text-books. The causes are many, but chief among them are malnutrition and subinvolution. The symptoms that first attract the attention of patient and physician are leucorrhœa and disordered menstruation, and if the disease is not arrested will result in graver complications, such as cystitis, vaginitis and hyperplasia, the latter causing displacements of the uterus, by substituting for the dense framework of that organ, a flaccid areolar tissue incapable of supporting it. These changes occur after cystic degeneration of the cervical mucous glands.

A diagnosis is easily made—the thick, tenacious mucus exuding from the os, a puffed or roughened granular os may be felt, or the mucous glands alone may be involved. It is important to decide whether or not we have to deal with corporeal endometritis as well. The disease rarely, if ever, gets well unaided.

TREATMENT: Besides the general regimen, which is of great importance, you want alterative applications to the cervix in mild or recent cases; incisions or dilatation and curetting in graver ones. In this disorder the affected parts can be reached with ease, and it would be reasonable to suppose that we ought to cure it without difficulty with local applications, and such has been my experience in a large number of cases; but in all cases of severe glandular disease nothing short of absolute destruction of the glands with a curette will relieve it.

The next subject to engage our attention is corporeal endometritis, a disease of not so frequent occurrence as endocervicitis, certainly, but in my opinion much oftener met with than some authors would lead us to believe. This, like endocervicitis, is a glandular disease, the metricular follicles being the seat of trouble and source of the characteristic leucorrhœal discharge, a thin muco-serous or muco-purulent secretion. In long standing cases the utricular glands atrophy, and large numbers become obliterated, involving the destruction of portions of mucous membrane, which is replaced by thin connective tissue, the cavity of the uterus is nearly always enlarged and walls thinner, unless complicated by arcolar hyperplasia or subinvoluion, the mucous membrane swollen, soft and smooth, or covered with granulations; bleeds freely when touched with a probe.

The chief causes are subinvolution, sudden checking of the menstrual flow, obstructions to the flow from whatever cause, displacements, etc.

Diagnosis is not very difficult; the leucorrhœa, enlarged cavity, bleeding upon the slightest touch, throbbing, bearing down in the supra-pubic region, and nearly always sterility. The reflex neuroses are very much the same for the various uterine disorders.

TREATMENT: Local applications in this disease except, perhaps, in very mild or any recent cases, are nearly useless, and certainly do not effect a cure, and for the best of reasons. In the first place it is impossible to thoroughly apply your remedies to the diseased lining, either with solid caustics or with pledgets of cotton dipped in a medicated solution, on account of its inaccessibility. Most physicians use pledgets of cotton when nearly all the solution is pressed out, as it passes through the cervix. The only possible way to reach the entire lining with your applications would be to dilate the cervix, generally under an anæsthetic, and confine the patient in bed for some time afterward; a thing that would prove impracticable, if it had to be repeated very often.

Intra-uterine injections, condemned by some and feared by nearly all, is the only possible means of making applications to every part of the mucous lining, without dilatation; and I only regret that my experience has not been large enough to speak of them with greater confidence, for I have used them many times without a single accident of any consequence. My resorting to them in the first instance was somewhat accidental. During my early practice, before I had given the subject of gynecology very much study, or had a very clear idea of the pathological conditions causing the trouble, I was called to treat some cases of uterine hemorrhage in the non-puerperal state. As these cases grew somewhat alarming, and the hemorrhage increased after each attempt to make astringent applications, I decided to try injections of carbolic and of tannic acids, which not only arrested the hemorrhage but cured my patients as well, or at least they thought themselves well at the time, which contributed not a little to my reputation, as other physicians had failed to afford any relief; so, after such an experience, you can readily understand how I could persuade myself that the dangers of the method had been exaggerated, and to use a remedy that had proven so efficient. I therefore began to use them in intra-uterine disorders generally, but more especially where there was hemorrhage or much leucorrhœa; and in one case only did I have the slighest trouble, and that one, a not very severe uterine colic, yielded readily to hot applications over uterus and a hypodermic of morphine.

With the greatest possible precautions, in careful hands, with suitable instruments provided with return flow, and carefully inspected before using, with gentle pressure on, what I prefer, the rubber bulb, and watching carefully to see that the return flow responds sufficiently to each pressure, I repeat, that if all of these precautions are taken, I believe the practice to be justifiable and safe.

In serious, cases, however, the most efficient, safe and expeditious manner of dealing with them is with the sharp curette.

Now, gentlemen, in regard to curetting, you often hear its advocates say that they do in a day what it took gynecologists, in former times, months to accomplish. Now, in a certain sense this is true, but not in the one you are very likely to take it in. I have been using these instruments for fifteen years, not with the same boldness that I now do I must admit, for I was taught by Thomas that you could cut, scrape or cauterize the cervix with impunity, but that it was decidedly dangerous to handle the body in any such manner. More recently, believing that I had used unnecessary caution, I have boldly attacked the endometrium with the sharp curette; and that there be no fault in the technique of my operations, I have talked personally with eminent gynecologists who report excellent results, but can discover no fault with my own methods. I treat the organ antiseptically afterwards, packing the cavity with antiseptic gauze; but the majority of my patients do not get well in a few days. Now a moment's thought will make you understand this. That they accomplish in a day what it took months to do with local applications, is correct in regard to the lining of the uterus. The removal of all vegetations, the rupturing of the turgid blood vessels in the mucous lining, and the destruction of all diseased glands, if it be done thoroughly, will cure the endometritis in a short time, and is unquestionably the correct treatment; but in the great majority of cases your patient is not cured. There are a number of complications, such as cystitis, rectitis, uterine displacements, areolar hyperplasia, etc., some of them, it may be, results of the disease in question, that must yet be cured and will require time.

The object of my remarks is not in any way to discredit the curette, but to put you on your guard against expecting too much from it, and to remind you that you still have other morbid symptoms to meet after the trouble, which it is especially intended to cure, has been relieved.

As I said at the beginning of this paper, I will briefly notice areolar hyperplasia, because it is a complication that so frequently persists after you have cured your endometritis, continuing a number of distressing symptoms on account of the increased size and weight of the uterus and resulting displacements. This disease, until recently, was thought to be a chronic inflammation of the uterine parenchyma, the so-called "chronic metritis."

More recently the microscope has brought to light the fact that it is a proliferation of the tissue, principally connective, due to chronic hyperæmia, which hyperæmia is kept up by interference with the retrograde metamorphosis of the puerperal uterus, called subinvolution, also by endometritis, lacerated cervix, malpositions, etc. The cervix, being more liable to mechanical irritation, is more often affected.

One symptom of importance is the amount of pain during sexual intercourse; but the physical signs are of most value from a diagnostic standpoint. The uterus is very large and heavy, especially the cervix, which hangs low down in the pelvis and is very tender to the touch. Many serious disorders accompany this disease, either as cause or effect, such as cystitis, rectitis, cellulitis, etc.

TREATMENT:—Cure the endometritis or endocervicitis, if they are complications—remove any vegetations that may be on the mucous lining, or granulations from the cervix; make free incisions into the swollen cervix; deplete with cotton and glycerine, use vaginal douches, and make intrauterine applications, such as carbolic acid and iodine; but more especially support the organ and correct the malpositions.

